# Tailoring Mechanical and Magnetic Properties in Dual-Phase FeCoNi(CuAl)_0.8_ High-Entropy Alloy

**DOI:** 10.3390/ma16227222

**Published:** 2023-11-18

**Authors:** Xiaohua Tan, Lingmiao Chen, Mengxin Lv, Wenfeng Peng, Hui Xu

**Affiliations:** Institute of Materials, School of Materials Science and Engineering, Shanghai University, Shanghai 200444, China; flippedclm@163.com (L.C.); lvmengxin1998@163.com (M.L.); wen22722361@163.com (W.P.)

**Keywords:** dual-phase high-entropy alloy, nanoindentation, magnetic property, mechanical property, first-principles calculations

## Abstract

For tailoring the mechanical and magnetic properties of dual-phase high-entropy alloys (HEAs), it is crucial to understand the effect of each phase on the overall properties. In this paper, the effects of individual FCC and BCC phases on the mechanical and magnetic properties of the FeCoNi(CuAl)_0.8_ HEA are investigated by nanoindentation and first-principles calculations. The nano-hardness of the BCC phase is 8.73 GPa, which is nearly double the 4.60 GPa of the FCC phase, which ascribes to spherical nanoprecipitates that are only observed in the BCC phase leading to precipitation hardening. First-principles calculations on the electronic structure show that calculated saturation magnetization (M_s_) of the BCC phase is 0.81 T, higher than 0.77 T of the FCC phase. An approximate yield strength and M_s_ can be estimated by summing the volume-fraction-weighted contributions from each phase, and are in good agreement with experimental values. It indicates that the overall mechanical and magnetic properties of the dual-phase HEAs can be tailored by tuning the volume fraction of the individual phase. Our findings are helpful to design prospective dual-phase HEAs with both good mechanical properties and soft magnetic performance by adjusting the content of each phase.

## 1. Introduction

A new alloying strategy of multi-principal-element alloys or high-entropy alloys (HEAs) was proposed in 2004 [1,2]. These alloys typically consist of more than five principal elements with equal or near-equal ratios and prone to form a solid solution with a simple body-centered cubic (BCC), face-centered cubic (FCC), or hexagonal close-packed (HCP) structure. Combined with four core effects (i.e., high entropy effect, lattice distortion, sluggish diffusion, and cocktail effect), HEAs exhibit excellent properties such as high strength and ductility, high fracture and fatigue resistance [3,4,5,6,7,8,9]. However, in single-phase HEAs, the presence of strength-ductility trade-off restricts their engineering applications. For example, HEAs with an FCC structure show high ductility and limited strength, while BCC-structured HEAs display high strength and unsatisfactory plasticity [10]. Hence, developing dual-phase HEAs is a prospective strategy to overcome the strength-ductility trade-off. For example, Raabe D. et al. used a metastability-engineering strategy to obtain excellent strength–ductility combinations in dual-phase Fe-Mn-Co-Cr HEAs [11]. Other studies employed phase transformation and optimized microstructure to have benefits of both the ductile FCC phase and hard BCC phase, and subsequently yields high strength and ductility [12,13,14,15].

It is worth noting that the complexities at small length scales including nanoprecipitates are observed in some dual-phase HEAs, which indicates that the generally used measurement at macroscopic scales cannot give deeper insights on the overall mechanical behavior of dual-phase HEAs. As a useful technique of measuring small scales, the nanoindentation can provide quantitative details about mechanical properties of HEAs including plasticity, hardness, and fracture toughness at the micro- and nano-scales [16,17,18,19,20,21,22,23]. Cui et al. studied mechanical properties of the CoFeNiMnTi_0.5_Al_0.5_ HEA by nanoindentation and found that the nano-hardness of the BCC phase was more than two times that of the FCC phase [24]. Cheng et al. also found that the nano-hardness of the BCC phase was larger than that of the FCC phase in the CoCrCuFeNi(Ti, B_4_C)_0.5_ HEA [25]. Sun et al. used nanoindentation and stated that the BCC phase had a stronger solid-solution strengthening leading to a higher hardness than the FCC phase in the Al_x_CoCrCuFeNi HEAs [26], while Zhang et al. found that the nano-hardness of the BCC phase in the (CoCrFeNi)_6−x−y_Cr_x_Al_y_ HEAs was one times higher than that of the FCC phase due to dominated precipitation strengthening [27]. This indicates that the underlying mechanical mechanism of individual FCC or BCC phases is not consistent and needs to be further investigated.

The ferromagnetic dual-phase (FCC + BCC) HEAs are expected to become candidates of novel soft magnetic materials because of a combination of good soft magnetic properties and mechanical properties [28,29]. However, the ever-increasing demand for advanced soft magnetic materials to operate under vibration or high-speed rotation conditions requires them to improve magnetic and mechanical properties. Hence, it is crucial to understand the effect of individual FCC and BCC phases on the overall magnetic and mechanical properties, and subsequently design dual-phase HEAs with both excellent soft magnetic performance and mechanical properties. The Fe-Co-Ni-Cu-Al-based HEAs have attracted much more attention due to having multi-properties such as good corrosion resistance, electromagnetic properties, good soft magnetic and mechanical properties [30,31,32,33]. Our previous work found that the FeCoNi(CuAl)_0.8_ HEA exhibited good soft magnetic and mechanical properties at room temperature [34,35,36]. However, the influence of individual FCC and BCC phases on the mechanical and magnetic properties is still not fully determined. In this work, we investigate the local mechanical properties of the FeCoNi(CuAl)_0.8_ HEA by nanoindentation at room temperature to shed insights on the effect of each phase on the overall mechanical properties. Moreover, the electronic structure is calculated by first-principles calculations to depict the contribution of individual FCC and BCC phases on the magnetic property. Finally, the volume-fraction-weighted contributions from each phase on overall mechanical and magnetic properties are explored.

## 2. Materials and Methods

The FeCoNi(CuAl)_0.8_ (Fe_21.74_Co_21.74_Ni_21.74_Cu_17.39_Al_17.39_ in at%) alloy was prepared by arc melting a mixture of pure metals Fe (99.98%), Co (99.999%), Ni (99.995%), Cu (99.999%), Al (99.99%) in an argon atmosphere. Before melting the alloys, the furnace chamber was evacuated and followed by flushing and backfilling with high-purity argon gas. The Ti-getter was placed inside the chamber to remove unwanted gas including nitrogen and oxygen. The alloy buttons were flipped and remelted six times to ensure chemical homogeneity. The rod-like samples with a size of 2 mm in diameter and 45 mm in length were prepared by sucking the molten alloy into a water-cooled copper mold; see Figure S1 in Supplementary Materials. The nanoindentation measurements at room temperature were carried out by a KIA Imicro nanoindentation instrument with a Berkovich diamond tip (KLA Corporate, Milpitas, CA, USA). The strain rate was 0.2 s^−1^ and the maximum load on the mirror-surface of samples was 20 mN with holding time of 5 s. The values of hardness and Young’s modulus were obtained from indentation load-depth curves [37,38]. The saturation magnetization was measured at room temperature by a Lake Shore 7407 vibrating sample magnetometer (VSM, Lake Shore Cryotronics, Westerville, OH, USA) with an applied field of 1.8 T. The X-ray diffraction (XRD) patterns from 20° to 100° with a scanning rate of 1° per min were recorded using a D/max-2550 diffractometer with Cu-Kα radiation (Rigaku Corporation, Akishima-Shi, Japan). The backscattered electron (BSE) and electron backscatter diffraction (EBSD) images were measured by a Tescan mira3 scanning electron microscope (SEM) with an EBSD detector (Bruker e-FlashFS, Bruker Nano GmbH, Hamburg, Germany). During the EBSD experiment, the acceleration voltage was 20 kV and the step size was 2 μm. The EBSD data were analyzed by the software of Esprit 2.1. The samples for EBSD measurement were ground by abrasive papers to mirror-surface and electro-polished in a solution of 10% HClO_4_ + 90% ethanol under the voltage of 29 V. A transmission electron microscope (TEM) (JEM-2100F, JEOL Ltd., Tokyo, Japan) was used to observe the microstructure. The TEM samples were prepared by a Helios 600i focused ion beam (FIB, FEI Corporate, Hillsboro, OR, USA). First-principles calculations were employed to calculate the electronic structure using the Vienna ab initio simulation package (VASP) [39]. The Perdewe-Burkee-Ernzerhof (PBE) function of the generalized gradient approximation (GGA) was used to treat electron exchange and correlation [40]. The solid solution models of FCC and BCC structures in HEAs were established using the virtual crystal approximation (VCA) [41]. The Brillouin-zone integrations were performed by the Monkhorst-Pack k-point meshes [42]. The energy cutoff was a constant of 400 eV in calculations.

## 3. Results

### 3.1. Microstructure

**Figure 1:**
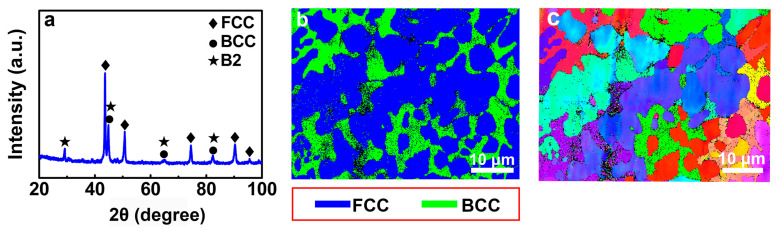
The microstructure of the FeCoNi(CuAl)_0.8_ HEA. (**a**) XRD pattern; (**b**) The phase map shows the presence of FCC phase and BCC phase; (**c**) The inverse pole figure map shows that the grains are randomly distributed.

**Figure 2:**
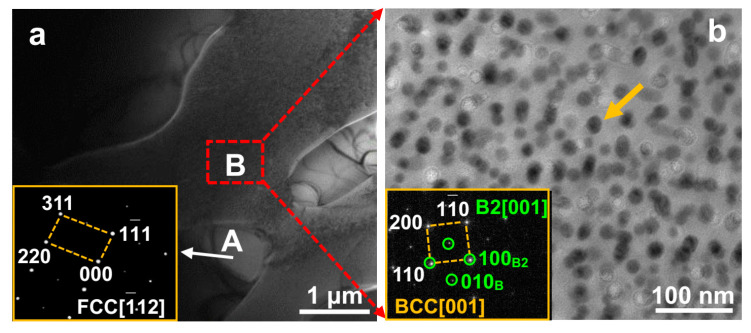
(**a**) The TEM bright-field image of the FeCoNi(CuAl)_0.8_ HEA, and the inset is SAED pattern of region A; (**b**) The enlarged image of region B, and the inset is SAED pattern. The nanoprecipitate is marked as an arrow.

The XRD pattern in Figure 1a shows that the FeCoNi(CuAl)_0.8_ HEA consists of the FCC phase, BCC phase, and B2 phase. The phase map measured by EBSD only shows the presence of the FCC phase (blue color) and BCC phase (green color). The volume fraction of each phase is obtained from the phase map in Figure 1b, that is, 74.0% FCC and 26.0% BCC. The average grain size is 8.36 μm, which is determined from the EBSD measurement. The B2 phase is observed by TEM (see Figure 2). Figure 1c is an inverse pole figure (IPF) map, and the grains are randomly distributed in the sample.

Figure 2a shows a bright-field TEM image of the FeCoNi(CuAl)_0.8_ HEA including A and B regions. Region A is identified as the FCC phase by indexing the selected area electron diffraction (SAED) pattern in an inset of Figure 2a. No precipitate is observed in the FCC phase (region A), while spherical precipitates with an average size of 18 ± 1 nm are visible in region B (see the enlarged image of region B in Figure 2b). The matrix is indexed as a BCC structure, and the B2 nanoprecipitate is confirmed by indexing the superlattice (marked as solid-circle pattern) in the inset of Figure 2b. Moreover, the B2 structure has a coherent relationship with the BCC matrix, that is, (110)_B2_‖(110)_BCC_ and [001]_B2_‖[001]_BCC_. Moreover, nanoprecipitates with BCC and face-centered tetragonal (FCT) structures are also observed and shown as Figure S2 in Supplementary Materials.

### 3.2. Local Mechanical Property

**Figure 3:**
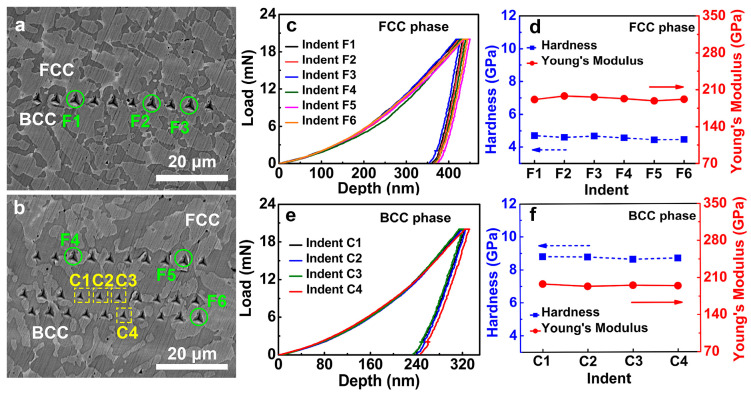
(**a**,**b**) Nanoindentation arrays of forty indents in the BSE images of the FeCoNi(CuAl)_0.8_ HEA, six indents of FCC phase are marked as solid circles and labeled as F1–F6, four indents of BCC phase are marked as dash square and numbered as C1–C4; (**c**–**f**) The load-depth (p–h) curves, nano-hardness and Young’s modulus as a function of indents of the FCC phase and the BCC phase. The respective solid arrow and dashed arrow represents corresponding Y-axis.

The nanoindentation arrays with forty indents in the FeCoNi(CuAl)_0.8_ HEA are shown in the BSE images in Figure 3a,b. Six indents of the FCC phase are chosen (marked as solid circles and labeled as F1–F6), and four indents of the BCC phase are marked as dashed square and numbered as C1–C4. The remaining indents are not completely located on the FCC phase or BCC phase and not considered in this work. The overlapped six nanoindentation load-depth (p–h) curves of the FCC phase in Figure 3c and minor fluctuation of hardness and Young’s modulus in Figure 3d indicate good nanoindentation repeatability of the FCC phase. The nanoindentation result of the BCC phase also exhibits reliable data, as shown in Figure 3e,f.

**Figure 4:**
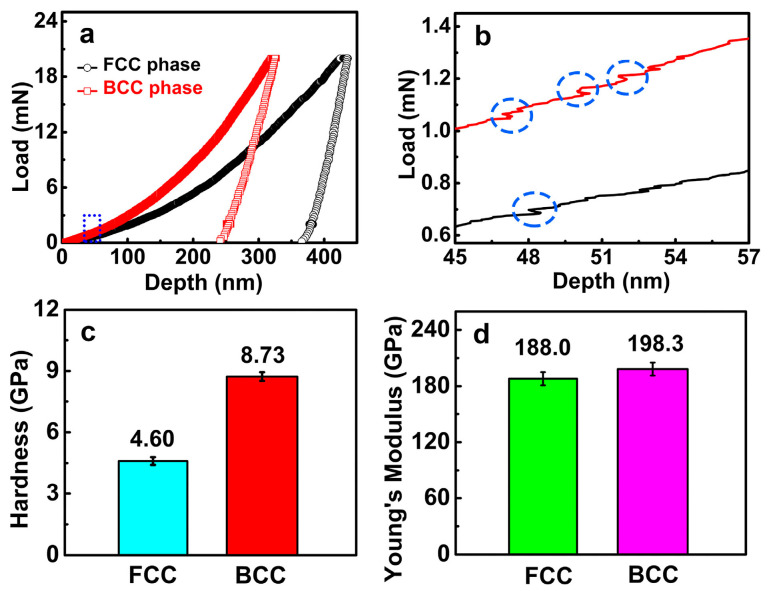
(**a**) Nanoindentation load-depth (p–h) curves of FCC phase and BCC phase; (**b**) The enlarged p–h curves of a dashed rectangle in (**a**), small serrations resulting from noise are marked as dashed circles; (**c**,**d**) the average value of hardness and Young’s modulus of FCC phase and BCC phase in the FeCoNi(CuAl)_0.8_ HEA.

In order to have a clear comparison, Figure 4a shows p–h curves of the FCC phase and BCC phase of the FeCoNi(CuAl)_0.8_ HEA. The indentation depth of the FCC phase is 430.0 nm, larger than 320.0 nm of the BCC phase, which indicates that the BCC phase has a higher resistance ability to plastic deformation than the FCC phase. Figure 4b shows enlarged p–h curves of a dash rectangle in Figure 4a and show some small serrations (marked as dashed circles). It is worth noting that these serrations are not from the phenomenon of “pop in”, but result from the noise of nanoindentation facility; see detailed descriptions about Figures S3 and S4 in Part I in Supplementary Material. Figure 4c shows that the average nano-hardness of the BCC phase is 8.73 ± 0.10 GPa, 1.90 times larger than the 4.60 ± 0.08 GPa of the FCC phase. The nano-hardness in this work is higher than the values reported in the dual-phase CoFeNiMnTi_0.5_Al_0.5_ HEA and CoCrCuFeNi(Ti,B_4_C)_0.5_ HEA [24,25]. The minor change of Young’s modulus in Figure 4d is observed with 188.0 ± 2.0 GPa of the FCC phase and 198.3 ± 2.6 GPa of the BCC phase. They are comparable to reported Young’s modulus in the dual-phase CoFeNiMnTi_0.5_Al_0.5_ HEA [24], but lower than the Al_1.5_CoCrCuFeNi HEA and AlCoCrFeNi HEA [26,43].

On the basis of above results of the micro-mechanical properties of individual FCC and BCC phases, an approximate yield strength of the dual-phase FeCoNi(CuAl)_0.8_ HEA can be obtained by summing the volume-fraction-weighted contributions to strength from each phase. In this work, the TEM result in Figure 2 shows that no precipitate is observed in the FCC phase, while nanoprecipitates are visible in the BCC phase. Hence, the estimated yield strength (*σ*) is expressed by [44],

(1) $$\sigma =f_{FCC}\;\sigma_{FCC}+f_{BCC}\;(\sigma_{BCC}+∆\sigma_{BCC})$$

where *f_FCC_* and *f_BCC_* are the volume fraction of the FCC phase and BCC phase, respectively, *σ_FCC_* and *σ_BCC_* are the respective yield strengths of the FCC phase and BCC phase due to solid-solution hardening, *Δσ_BCC_* is the increase of the yield strength due to the nanoprecipitates.

It is found from the TEM result in Figure 2 that the small nanoprecipitates (18 ± 1 nm) are coherent with the matrix, indicating they can impede dislocation motions by a particle shearing mechanism [45]. Hence, the *Δσ_BCC_* in Equation (1) is mainly from order strengthening (*Δσ_order_*), modulus strengthening (*Δσ_modulus_*), coherency strengthening (*Δσ_coherency_*), and can be given by

*Δσ*_*BCC*_ = *Δσ*_*order*_ + *Δσ*_*modulus*_ + *Δσ*_*coherency*_ (2)

The calculated descriptions about the *σ_FCC_*, *σ_BCC_*, and *Δσ_BCC_* are shown in Part II in Supplementary Material. In this work, the respective volume fraction of the FCC phase (*f_FCC_* = 74.0%) and BCC phase (*f_BCC_* = 26.0%) is obtained from EBSD result in Figure 1. The values of *σ_FCC_*, *σ_BCC_*, and *Δσ_BCC_* are about 216.0 MPa, 917.0 MPa, and 624.0 MPa. Using Equation (1) and these parameters, the calculated yield strength of the FeCoNi(CuAl)_0.8_ HEA is about 560.0 MPa, which is close to the experimentally measured value of 537.0 MPa from compressive engineering stress-strain curves (see Figure S5 in Supplementary Material).

Young’s modulus is a measure of the resistance to separation of adjacent atoms and depends on the type of interatomic binding forces. In the FeCoNi(CuAl)_0.8_ HEA, the metallic bonds dominate. The minor change of Young’s modulus about FCC and BCC is observed, which can be explained from two aspects. (1) The effect of the crystal structure. The FCC structure has a denser atomic packing than the BCC structure [46]. It indicates that, in the FCC structure, the reduction of the interatomic distance and the enhancement of the interatomic binding force in the FCC structure may lead to the increase of Young’s modulus. (2) The effect of the microstructure. The TEM result shows that no precipitate is observed in the FCC phase, while nanoprecipitates are visible in the BCC phase. The presence of precipitates can cause the lattice distortion and strain field between precipitates and matrix leading to the increase of Young’s modulus [47,48,49]. That is, the first effect about the crystal structure is beneficial to increasing Young’s modulus of FCC, while the second one is helpful to improve Young’s modulus of BCC. The two roles result in the minor change of Young’s modulus about FCC and BCC.

### 3.3. Magnetic Property

**Figure 5:**
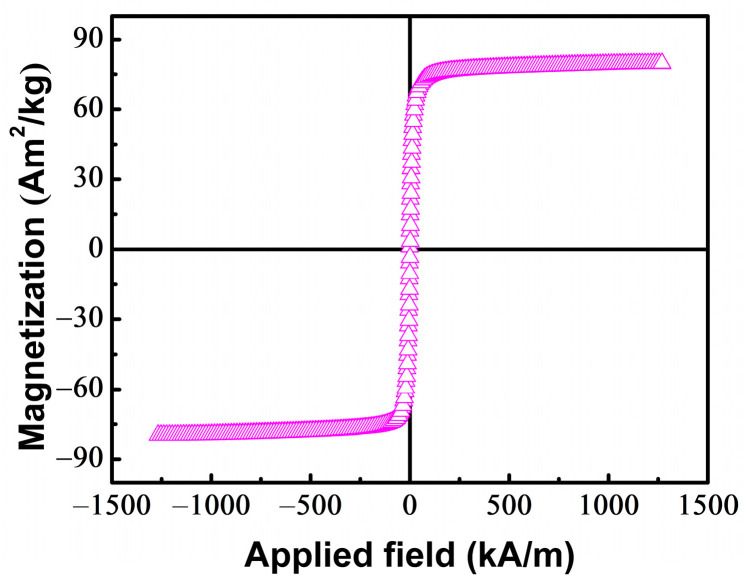
The hysteresis loops of the FeCoNi(CuAl)_0.8_ HEA measured at room temperature.

**Figure 6:**
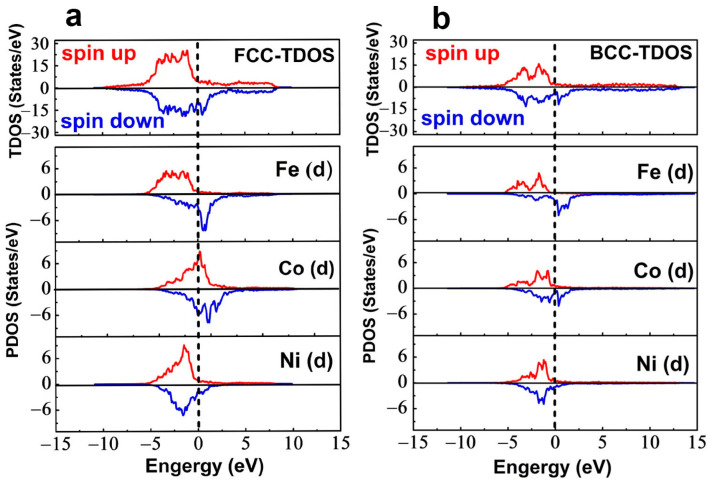
(**a**,**b**) Total density of states (TDOS) and partial density of states (PDOS) of FCC and BCC in the FeCoNi(CuAl)_0.8_ HEA. The Fe (d), Co (d), and Ni (d) represents d-orbital density of states of Fe, Co, Ni metals. The upper and lower curves show spin-up and spin-down states, respectively. The Fermi level is set at zero energy and indicated by a vertical dashed line.

Figure 5 shows the hysteresis loop of FeCoNi(CuAl)_0.8_ HEA at room temperature. It exhibits soft magnetic behavior with the saturation magnetization (*M_s_*) of 78.9 ± 2.2 Am^2^/kg (≈0.75 T). The total density of states (TDOS) of FCC and BCC in the FeCoNi(CuAl)_0.8_ HEA is shown in Figure 6. The Fermi level is set at zero energy and is indicated by vertical dashed lines. Since the d-orbital DOS of Fe, Co, Ni metals plays a crucial role in their physical properties, we project out the contribution from the d orbitals within Fe, Co, Ni and term them Fe (d), Co (d), Ni (d) in the partial density of states (PDOS). It is found that the BCC has larger magnetization than FCC, which can be explained from four main differences of TDOS between FCC and BCC in Figure 6. (1) The distribution of spin-up and spin-down in TDOS is not symmetric, leading to a ferromagnetic configuration [50,51]. Bigger asymmetric spin-up and spin-down distribution is observed in BCC, resulting in yielding larger magnetic moment than that of FCC. (2) The spin-up density of states (DOS) of BCC shows sharp d-band edges, resulting in a large DOS at the Fermi level, which is conducive to a large magnetization. (3) The upper edge of the spin-up TDOS of FCC in Figure 6a becomes rounded relative to that of BCC in Figure 6b, which indicates that the d band of FCC is filled and becomes fuller, leading to a reduction of the magnetization [52]. (4) In Figure 6a, the PTOS of Co (d) shows the upper peak of the majority-spin at Fermi level, indicating that Co (d) bands are partially occupied. It can decrease the magnetic moment and weaken ferromagnetic behavior of FCC [53]. 

Our previous work found that the BCC structure showed higher saturation magnetization than the FCC structure by semi-quantitative analysis [36]. In this work, we can calculate the saturation magnetization of individual FCC and BCC based on TDOS results. The saturation magnetization, *M_s_*, can be calculated by [46]

(3) $${\;M}_{s}=\frac{n_{B}\mu_{B}}{V_{C}}$$

where *μ_B_* is Bohr magneton, *n_B_* is Bohr magnetons per unit cell, *V_C_* is the unit cell volume. 

The values of *n_B_* and *V_C_* can be obtained from the TDOS result. Using Equation (3), the saturation magnetization of the FCC phase (*M*_*s*(*FCC*)_) and BCC phase (*M*_*s*(*BCC*)_) is estimated as 0.77 ± 0.02 T and 0.81 ± 0.02 T. Hence, the total *M_s_* of the dual-phase FeCoNi(CuAl)_0.8_ HEA can be approximately estimated by summing the volume-fraction-weighted contributions to *M_s_* from each phase as follows:

(4) $$M_{s\;(total)}=M_{s\;(FCC)}V_{FCC}+M_{s\;(BCC)}V_{BCC}$$

The *V_FCC_* and *V_BCC_* is the volume faction of the FCC phase (74.0%) and BCC phase (26.0%). Therefore, the *M*_*s*(*total*)_ of the FeCoNi(CuAl)_0.8_ HEA is estimated as 0.78 ± 0.02 T, which is comparable to the experimentally measured value of the saturation magnetization (*M_s_* = 0.75 ± 0.02 T).

The enlightenment of this work is that the mechanical and magnetic properties can be improved by tuning the volume fraction of FCC and BCC in dual-phase HEAs. Considering the higher *M_s_* and larger contribution on the yield strength of the BCC phase, we can increase the fraction of the BCC phase by increasing the content of (CuAl) and design prospective dual-phase HEAs with both good mechanical and magnetic properties. Hence, we prepared dual-phase FeCoNi(CuAl)_x_ (x = 0.9, 1.0) HEAs with more volume fraction of the BCC phase and measured their mechanical and magnetic properties at macroscopic scales. Table 1 lists the values of the yield strength (*σ*_0.2_) and plastic strain (*ε*) that are obtained from compressive engineering stress-strain curves of FeCoNi(CuAl)_x_ (x = 0.8, 0.9, 1.0) HEAs. The experimental values of the saturation magnetization (*M_s_*) are also given in Table 1. It is found that the fraction of the BCC phase is increased with increasing the content of (CuAl). In comparison to the FeCoNi(CuAl)_0.8_ HEA, the yield strength and saturation magnetization are both improved in the FeCoNi(CuAl)_x_ (x = 0.9, 1.0) HEAs due to the presence of more fraction of the BCC phase. The FeCoNi(CuAl)_1.0_ HEA consists of the highest fraction of BCC phase (89.0%) and exhibits the largest yield strength (~1500 MPa). The *M_s_*, however, is 0.79 T, which is lower than the 0.84 T of the FeCoNi(CuAl)_0.9_ HEA. It attributes to the diluted effect of the addition of non-magnetic Cu and Al. The respective estimated yield strength of x = 0.9 HEA and x = 1.0 HEA is 1375.0 MPa and 1474.0 MPa, which is comparable to the experimental value of 1392.0 MPa and 1500.0 MPa (see Figure S6 and calculated descriptions in Part III of Supplementary Materials).

In this work, the *M_s_* of dual-phase FeCoNi(CuAl)_x_ (x = 0.8–1.0) HEAs is in the range of 0.75–0.84 T, which is higher than the *M_s_* of soft magnetic ferrites (0.35–0.5 T) and comparable to Supermalloy (Ni_80_Fe_15_Mo_5_, *M_s_* = 0.8 T) [54]. Moreover, the dual-phase FeCoNi(CuAl)_x_ (x = 0.8 − 1.0) HEAs exhibit both good soft magnetic performance and mechanical properties, which can become candidates of advanced soft magnetic materials to meet the ever-increasing requirements for them to operate under high-speed rotation conditions and have prospective applications in transformers, motors, and generators under low frequency (<1 kHz) working conditions. Our findings provide an idea to design prospective dual-phase HEAs with both good mechanical and magnetic properties as advanced soft magnetic materials.

## 4. Conclusions

The effects of individual FCC and BCC phases on the microstructure, local mechanical properties, and magnetic properties of dual-phase FeCoNi(CuAl)_0.8_ HEA are investigated. The respective volume fraction of the FCC phase and BCC phase obtained from EBSD is 74.0% and 26.0%. The TEM result shows that no precipitate is shown in the FCC phase, while spherical B2 nanoprecipitates are observed in the BCC phase. The measurement of nanoindentation shows that the nano-hardness of the BCC phase (8.73 ± 0.10 GPa) is nearly double the 4.60 ± 0.08 GPa of the FCC phase due to the presence of nanoprecipitates in the BCC phase. It is found from first-principles calculations on the electronic structure that the magnetic moment of BCC is larger than that of FCC. The calculated saturation magnetization of the FCC phase and BCC phase is 0.77 ± 0.02 T and 0.81 ± 0.02 T, respectively. 

On the basis of the effect of individual FCC and BCC phases on the overall mechanical and magnetic properties, the calculated yield strength and saturation magnetization of the FeCoNi(CuAl)_0.8_ HEA by summing the volume-fraction-weighted contributions from each phase is in good agreement with measured values. Our findings indicate that the overall mechanic and magnetic properties can be tuned by adjusting the fraction of FCC and BCC phases, which gives an idea to design prospective dual-phase HEAs with both good mechanical and magnetic properties.

## Figures and Tables

**Table 1:** The measured yield strength (*σ*_0.2_), plastic strain (*ε*), saturation magnetization (*M_s_*), and phase constitution with volume fraction of FCC and BCC in the FeCoNi(CuAl)_x_ (x = 0.8, 0.9, 1.0) HEAs.

Alloys	*σ*_0.2_ (MPa)	*ε* (%)	*M_s_* (T)	Phase Constitution
x = 0.8	537.0	47.8	0.75	FCC (74.0%) + BCC (26.0%)
x = 0.9	1392.0	7.7	0.84	FCC (13.0%) + BCC (87.0%)
x = 1.0	1500.0	6.6	0.79	FCC (11.0%) + BCC (89.0%)

## Data Availability

The data presented in this study are available on request from the corresponding author.

## Supplementary Materials

The following supporting information can be downloaded at: https://www.mdpi.com/article/10.3390/ma16227222/s1, Figure S1: (a) Schematic illustration of copper mold casting. Number 1, 2, and 3 represents copper mold, water-cooled system, and melting metals; (b) The photo of rod sample with a size of 2 mm in diameter and 45 mm in length. Figure S2: (a,b) the HRTEM images of nanoprecipitates, and the insets are fast Fourier transformation indexed as BCC and FCT structures. Figure S3: Determination of instrumental noise. (a) Fitting curve of the depth as a function of time for the holding segment in the FeCoNi(CuAl)_0.8_ HEA; (b–f) the maximum Δh value (marked as red arrow) is measured during 4.6 s holding segment.; Figure S4: (a,b) The p–h curves (black line) and fitted curves (red line) of FCC phase and BCC phase in the FeCoNi(CuAl)_0.8_ HEAs; (a1,b1) the measured maximum difference (Δd, marked as red arrow) between the raw depth data and the fitted curve in the FeCoNi(CuAl)_0.8_ HEA.; Figure S5: The compressive engineering stress-strain curve of the FeCoNi(CuAl)_0.8_ HEA measured at room temperature. Figure S6: (a,b) The TEM bright-field images of the FeCoNi(CuAl)_x_ (x = 0.9, 1.0) HEAs. (c) The nanotwin is observed in the x = 1.0 HEA. Refs. [3,44,45,55,56,57,58,59,60,61,62,63,64] are cited in the supplementary materials.
