# Supplementary material for: Tailoring Mechanical and Magnetic Properties in Dual-Phase FeCoNi(CuAl)0.8 High-Entropy Alloy
Source: Materials (Basel). 2023 Nov 18;16(22):7222. doi: 10.3390/ma16227222 (PMC10672741; doi:10.3390/ma16227222)
Supplement: Supplementary file 1 [file materials-16-07222-s001.zip › materials-2702011-SI.pdf]

Supplementary Material

Tailoring mechanical and magnetic properties in dual-phase $FeCoNi(CuAl)_{0.8}$ high-entropy alloy

Xiaohua Tan*, Lingmiao Chen, Mengxin Lv, Wenfeng Peng, Hui Xu*

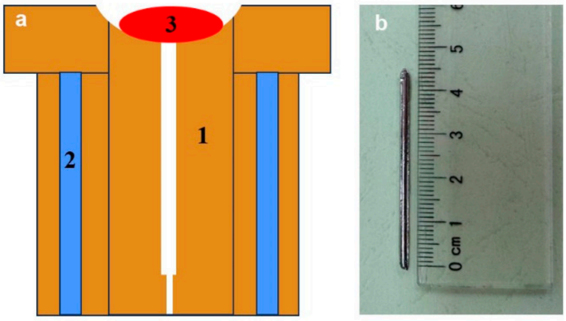

Figure S1. (**a**) Schematic illustration of copper mold casting. Number 1, 2, and 3 represents copper mold, water-cooled system, and melting metals; (**b**) The photo of rod sample with a size of 2 mm in diameter and 45 mm in length.

Figure. S2a and 2b are high-resolution transmission electron microscopy (HRTEM) images of nanoprecipitates. The structure of nanoprecipitation can be indexed as BCC and face-centered tetragonal (FCT) by fast Fourier transformation (FFT). The orientation relationship between FCT precipitate and BCC matrix displays Kurdjumov–Sachs (K-S) relationship, namely, $(11\bar{1})_{FCT}$ || $(011)_{BCC}$ and $[\bar{1}10]_{FCT}$ || $[1\bar{1}1]_{BCC}$.

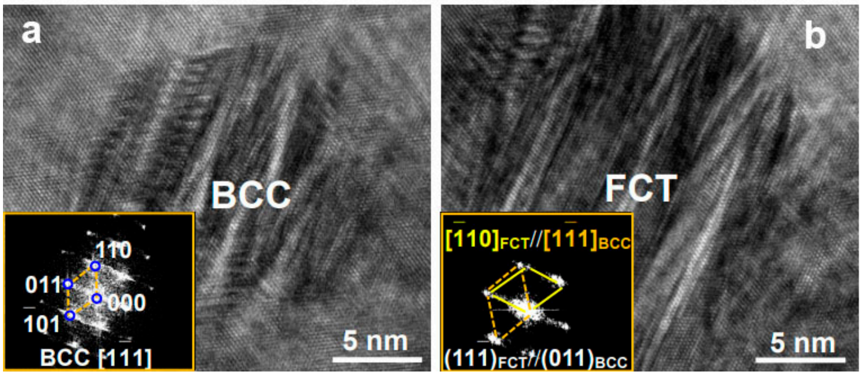

Figure S2. (**a, b**) the HRTEM images of nanoprecipitates, and the insets are fast Fourier transformation indexed as BCC and FCT structures.

Part 1

The analysis of serrations occurred in nanoindentation load-depth (p - h) curves

1. Determination of instrumental noise

In order to confirm the origin of the serrations in p - h curves, we chose experimental data of nanoindentation about the BCC phase in the $FeCoNi(CuAl)_{0.8}$ high-entropy alloy (HEA) as an example to determine the noise from the instrument.

The serrations from the background noise are extracted from the 4.6 s holding segment (from 50 s to 54.6 s). The depth as a function of time for the holding segment in the $FeCoNi(CuAl)_{0.8}$ HEA is shown in Figure S3a. Black points are experimental data and the red solid line is the fitting plot. In order to have a clear picture of serrations, we display 4.6 s holding segment in Figure S3b-f. The maximum Δh is the difference between the original depth data and the fitting curve (denoted as h_{raw} - h_{fit}). The maximum Δh value (marked as red arrow) is 2.55 nm, indicating that the background noise is 2.55 nm.

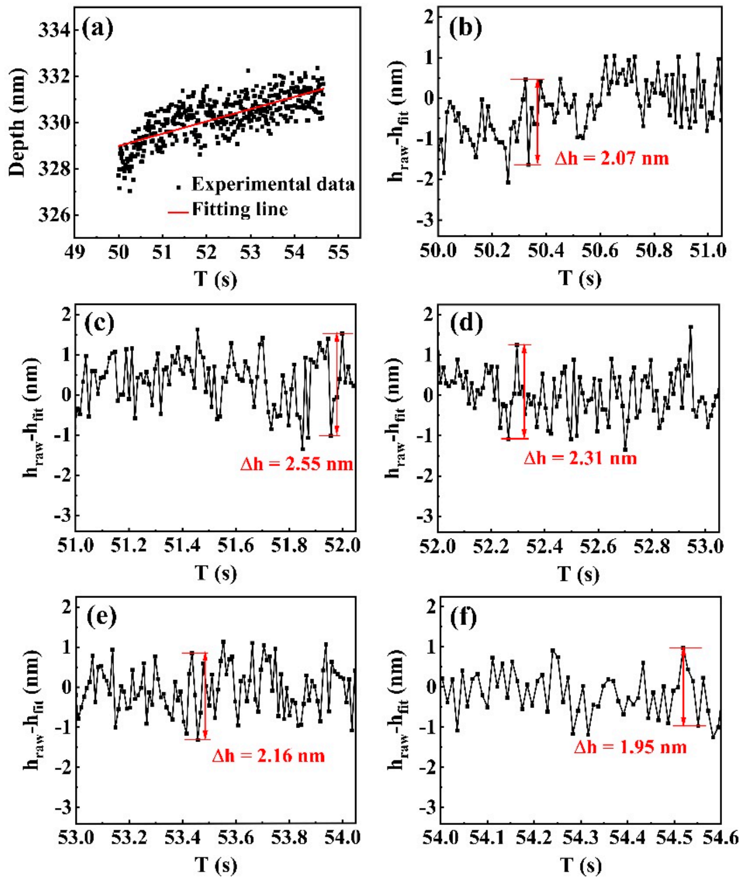

Figure S3. Determination of instrumental noise. (**a**) Fitting curve of the depth as a function of time for the holding segment in the $FeCoNi(CuAl)_{0.8}$ HEA; (**b-f**) the maximum Δh value (marked as red arrow) is measured during 4.6 s holding segment.

2. Determination of the origin of the serrations in p - h curves

Figure S4a and 4b are the p - h curves (black line) and fitted curves (red line) of the FCC phase and the BCC phase in the $FeCoNi(CuAl)_{0.8}$ HEA. The corresponding maximum difference (Δd, marked as red arrow) between the raw depth data and the fitted curve is 1.82 nm for the FCC phase and 1.02 nm for the BCC phase (see Figure S2a1 and 2b1). In comparison to the background noise (2.55 nm) in Figure S3, the values of Δd in Figure S4 are less than 2.55 nm, indicating that the serrations in p-h curves are from instrumental noise.

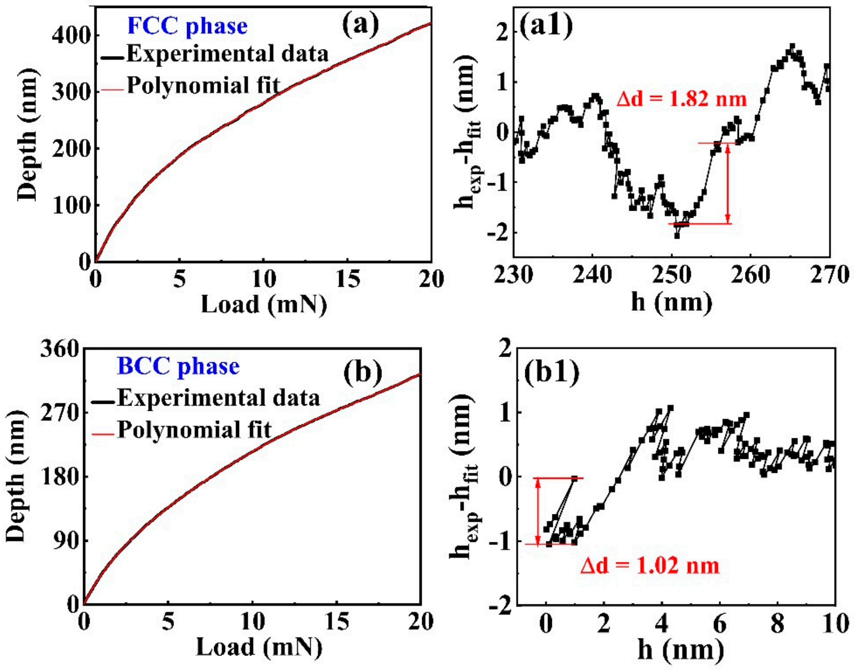

Figure S4. (a, b) The p-h curves (black line) and fitted curves (red line) of FCC phase and BCC phase in the $FeCoNi(CuAl)_{0.8}$ HEAs; **(a1, b1)** the measured maximum difference (Δd, marked as red arrow) between the raw depth data and the fitted curve in the $FeCoNi(CuAl)_{0.8}$ HEA.

Part II

The calcuated yield strength of the dual-phase $FeCoNi(CuAl)_{0.8}$ HEA

The strengthening mechanisms in this work mainly include solid-solution hardening and precipitation hardening. The yield strength of the $FeCoNi(CuAl)_{0.8}$ HEA can be approximately estimated as follows:

$$\sigma = f_{FCC}\,\sigma_{FCC} + f_{BCC}\,(\sigma_{BCC} + \Delta\sigma_{BCC}) \qquad (1)$$

where f_{FCC} and f_{BCC} are the respective volume fraction of FCC phase and BCC phase, $\Delta\sigma_{BCC}$ is the increase of the yield strength due to the precipitates, σ_{FCC} and σ_{BCC} are the respective yield strengths of FCC phase and BCC phase due to solid-solution hardening.

It is worth noting that how to evaluate the effect of solid-solution hardening between traditional alloys and HEAs has a distinct difference. Solid-solution strengthening in traditional alloys is based on the diluted effect in which solute elements with low concentration are distributed in the matrix of solvent elements. However, HEAs are concentrated solid-solution systems with equal or near-equal ratios and are difficult to define as “solute” and “solvent” elements. Although some models were reported to extend the traditional solid solution hardening theories to HEAs, they still have difficulties to describe HEAs with complex structures including precipitates and dual-phase structures [55-57]. That is, the precise evaluation of solid-solution strengthening in HEAs remains a challenge. In this work, the $FeCoNi(CuAl)_{0.8}$ HEA consists of a mixture of FCC and BCC, and nanoprecipitates embedded in the BCC phase. It indicates that the models in refs 55-57 might not give a precise evaluation of solid-solution strengthening in the individual FCC phase and BCC phase. Alternatively, we measured the yield strength of equal ratio FeCoNi HEA with single FCC phase (~216 MPa) and FeCoNiAl HEA with single BCC phase (~917 MPa) as the estimated value of the σ_{FCC} and σ_{BCC} in equation (1). This alternative method was used to estimate the yield strength in dual-phase medium-entropy alloys [44].

The nanoprecipitates in the BCC phase of the $FeCoNi(CuAl)_{0.8}$ HEA with the average size of 18 ± 1 nm are observed by TEM. Moreover, the nanoprecipitates are coherent with the matrix, which suggests that they impede dislocation motions by a particle shearing mechanism [5]. Hence, the increment of yield strength ($\Delta\sigma_{BCC}$) in equation (1) arising from dislocation cutting of the nanoprecipitates in the BCC phase is mainly from order strengthening ($\Delta\sigma_{order}$), modulus strengthening ($\Delta\sigma_{modulus}$), and coherency strengthening ($\Delta\sigma_{coherency}$). That is, the increment of yield strength, $\Delta\sigma_{BCC}$, can be expressed by

$$\Delta\sigma_{BCC} = \Delta\sigma_{order} + \Delta\sigma_{modulus} + \Delta\sigma_{coherency} \quad (2)$$

The expression of $\Delta\sigma_{order}$ is given by [45],

$$\Delta\sigma_{order} = 0.81M(\frac{\gamma_{APB}}{2b})(\frac{3\pi f}{8})^{\frac{1}{2}} \quad (3)$$

where M is the Taylor factor, γ_{APB} is the antiphase boundary energy, b is the Burgers vector for BCC, and f is the volume fraction of precipitates.

The value of b from measured lattice parameter is 0.2475, and the volume fraction of precipitates (f) is 29.6% in the FeCoNi(CuAl)$_{0.8}$ HEA. M is 2.73 for the BCC matrix [58], γ_{APB} is referred to in the literature [59] and taken as 200 mJ·m^{-2} for the precipitates in the BCC phase. Using equation (3) and these parameters, the order strengthening contribution can be estimated as 527.0 MPa.

The $\Delta\sigma_{modulus}$ is given by [60],

$$\Delta\sigma_{modulus} = 0.0055M(\Delta G)^{3/2}(\frac{2f}{Gb^2})^{1/2}b(\frac{r}{b})^{\frac{3m}{2}-1} \quad (4)$$

where ΔG is the difference of the shear modulus between the matrix and precipitates, G is the modulus of the matrix, r is the average value of radius of sphere precipitates, and m is a constant.

In the FeCoNi(CuAl)$_{0.8}$ HEA, the values of f, r, and b are 29.6%, 9.0 nm, and 0.2475. G is calculated from E/2(1+ν), E is Young's modulus obtained from nanoindentation data (198.3 GPa), ν is the Poisson's ratio and taken as 0.31 for BCC phase [61]. ΔG is taken as 3 GPa for BCC phase [62]. The value of m is 0.85, and M is 2.73 for BCC matrix [58]. Hence, the modulus strengthening contribution is estimated as 19.0 MPa.

The $\Delta\sigma_{coherency}$ is given by [63],

$$\Delta\sigma_{coherency} = M\alpha(G\varepsilon)^{3/2}(\frac{rf}{0.5Gb})^{1/2} \quad (5)$$

where α is constant and estimated as 2 [45]. The ε is the lattice mismatch between matrix and precipitates and is estimated by (2/3)× (Δa/a), where a is the lattice parameter of matrix. The coherency strengthening contribution is estimated as 78.0 MPa.

Based on above calculations and equation (2), the $\Delta\sigma_{BCC}$ is obtained with 624.0 MPa. The volume fraction of FCC phase (f_{FCC}) and BCC phase (f_{BCC}) can be obtained from EBSD result as 74% and 26%. Hence, the equation (1) is rewritten as,

$$\sigma = 0.74\,\sigma_{FCC} + 0.26\,(\sigma_{BCC} + \Delta\sigma_{BCC}) \quad (6)$$

The values of σ_{FCC} and σ_{BCC} are 216 MPa and 917 MPa. Hence, the yield strength of the FeCoNi(CuAl)$_{0.8}$ HEA is estimated as 560.0 MPa, which is comparable to the experimentally measured value of 537.0 MPa from compressive engineering stress-

strain curves (see Figure S5).

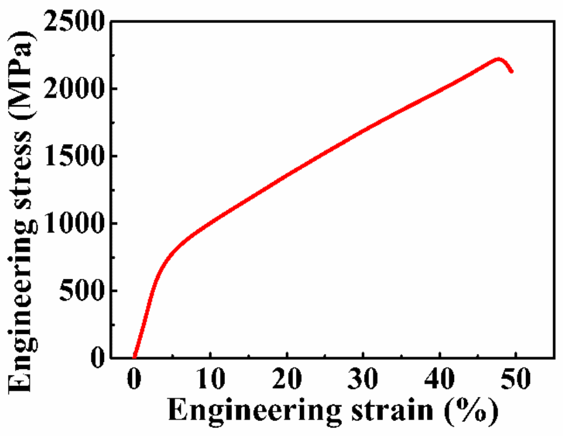

Figure S5. The compressive engineering stress-strain curve of the $FeCoNi(CuAl)_{0.8}$ HEA measured at room temperature.

Part III

(1) The calcuated yield strength of the dual-phase $FeCoNi(CuAl)_{0.9}$ HEA

The respective volume fraction of FCC phase (f_{FCC}) and BCC phase (f_{BCC}) in the $FeCoNi(CuAl)_{0.9}$ HEA (term as x = 0.9 HEA) is 13% and 87%. Hence, the yield strength of the x = 0.9 HEA can be approximately estimated as follows:

$$\sigma = 0.13\,\sigma_{FCC} + 0.87\,(\sigma_{BCC} + \Delta\sigma_{BCC}) \qquad (7)$$

The increment of yield strength, $\Delta\sigma_{BCC}$, can be expressed by

$$\Delta\sigma_{BCC} = \Delta\sigma_{order} + \Delta\sigma_{modulus} + \Delta\sigma_{coherency} \qquad (8)$$

In the x = 0.9 HEA, the Burgers vector (b) is 0.2476, the volume fraction of precipitates (f) is 31.0%, the modulus of the matrix (G) is 72.10 GPa, the average value of radius of sphere precipitates (r) is estimated as 10 nm from Figure S6(a), the lattice mismatch between matrix and precipitates (ε) is 0.0011. Using equations (3-5) and these parameters, the respective $\Delta\sigma_{order}$, $\Delta\sigma_{modulus}$, and $\Delta\sigma_{coherency}$ is 539.0 MPa, 20.0 MPa, and 72.0 MPa. That is, the value of $\Delta\sigma_{BCC}$ is 631 MPa. Hence, using equation (7), the estimated yield strength (σ) of the $FeCoNi(CuAl)_{0.9}$ HEA is 1375.0 MPa, which is comparable to the experimental value (1392.0 MPa).

(2) The calcuated yield strength of the dual-phase $FeCoNi(CuAl)_{1.0}$ HEA

In the $FeCoNi(CuAl)_{1.0}$ HEA (term as x = 1.0 HEA), the volume fraction of FCC phase (f_{FCC}) and BCC phase (f_{BCC}) is 11% and 89%, respectively. Hence, the yield

strength of the x = 1.0 HEA can be approximately estimated as follows:

$$\sigma = 0.11\,\sigma_{FCC} + 0.89\,(\sigma_{BCC} + \Delta\sigma_{BCC}) \quad (9)$$

The nanotwin is observed in the x = 1.0 HEA, as shown in Figure S6(c). Hence, the twin-boundary strengthening ($\Delta\sigma_{tb}$) is considered, and the increment of yield strength ($\Delta\sigma_{BCC}$) of the x = 1.0 HEA is expressed by

$$\Delta\sigma_{BCC} = \Delta\sigma_{order} + \Delta\sigma_{modulus} + \Delta\sigma_{coherency} + \Delta\sigma_{tb} \quad (10)$$

The $\Delta\sigma_{tb}$ can be given by [3]

$$\Delta\sigma_{tb} = f_t k_{tb} \lambda_t^{-\frac{1}{2}} \quad (11)$$

where f_t is the volume fraction of the grains with twins, k_{tb} is the twin-boundary strengthening coefficient, and λ_t is the average twin thickness.

In the x = 1.0 HEA, the Burgers vector (b) is 0.2478, the average value of radius of sphere precipitates (r) is 11.5 nm that is obtained from Figure S6(b), the volume fraction of precipitates (f) is 32.6%, the modulus of the matrix (G) is 67.33 GPa, the lattice mismatch between matrix and precipitates (ε) is 0.001, the value of λ_t is 2.63 nm. The value k_{tb} is estimated as 195 MPa·$\mu m^{1/2}$ [64].

Using equations (3-5), equation (11), and these parameters, the respective $\Delta\sigma_{order}$, $\Delta\sigma_{modulus}$, $\Delta\sigma_{coherency}$, and $\Delta\sigma_{tb}$ is 553.0 MPa, 22.0 MPa, 62.0 MPa and 76.0 MPa. Then the value of $\Delta\sigma_{BCC}$ is 713.0 MPa. Hence, using equation (9), the estimated yield strength (σ) of the $FeCoNi(CuAl)_{1.0}$ HEA is 1474.0 MPa, which is close to the experimental value (1500.0 MPa).

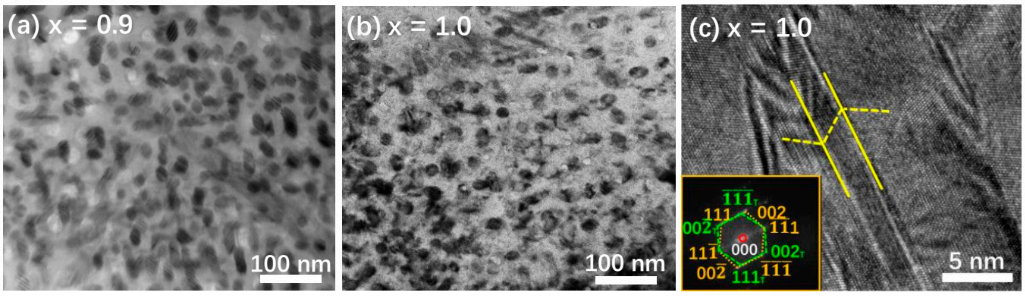

Figure S6. (**a, b**) The TEM bright-field images of the $FeCoNi(CuAl)_x$ (x = 0.9, 1.0) HEAs. (**c**) The nanotwin is observed in the x = 1.0 HEA.

Reference in Part II and Part III

[55] Senkov, O.N.; Scott, J.M.; Senkova, S.V.; Miracle, D.B.; Woodward, C.F. Microstructure and room temperature properties of a high-entropy TaNbHfZrTi alloy.

J. Alloys Compd. **2011**, *509*, 6043–6048.
[56] Toda-Caraballo, I.; Rivera-Díaz-del-Castillo, P.E.J. Modelling solid solution hardening in high entropy alloys. *Acta Mater.* **2015**, *85*, 14-23.
[57] Toda-Caraballo, I. A general formulation for solid solution hardening effect in multicomponent alloys. *Scr. Mater.* **2016**, *127*, 113–117.
[58] Rosenberg, J.M.; Piehler, H.R. Calculation of the Taylor factor and lattice rotations for bcc metals deforming by pencil glide. *Metall. Trans.* **1971**, *2*, 257–259.
[59] Gorbatov, O.I.; Lomaev, I.L.; Gornostyrev, Y.N.; Ruban, A.V.; Furrer, D.; Venkatesh, V.; Novikov, D.L.; Burlatsky, S.F. Effect of composition on antiphase boundary energy in Ni_3Al based alloys: Ab initio calculations. *Phys. Rev. B* **2016**, *93*, 224106.
[60] Tong, Y.; Chen, D.; Han, B.; Wang, J.; Feng, R.; Yang, T.; Zhao, C.; Zhao, Y.L.; Guo, W.; Shimizu, Y.; Liu, C.T.; Liaw, P.K.; Inoue, K.; Nagai, Y.; Hu, A.; Kai J.J. Outstanding tensile properties of a precipitation-strengthened $FeCoNiCrTi_{0.2}$ high-entropy alloy at room and cryogenic temperatures. *Acta Mater.* **2019**, *165*, 228-240.
[61] Hosford, W.F. Mechanical Behavior of Materials, 1st ed.; Cambridge University Press, 2005.
[62] ASM International Handbook committee, 2nd ed.; Properties and Selection: Irons, Steels and High Performance Alloys, ASM International, 1990.
[63] Ma, Y.; Hao, J.M.; Jie, J.C.; Wang, Q.; Dong, C. Coherent precipitation and strengthening in a dual-phase $AlNi_2Co_2Fe_{1.5}Cr_{1.5}$ high-entropy alloy. *Mater. Sci. Eng. A* **2019**, *764*, 138241.
[64] Su, J.; Raabe, D.; Li, Z.M. Hierarchical microstructure design to tune the mechanical behavior of an interstitial TRIP-TWIP high-entropy alloy. *Acta Mater.* **2019**, *163*, 40–54.
